# The Origin of the Variola Virus

**DOI:** 10.3390/v7031100

**Published:** 2015-03-10

**Authors:** Igor V. Babkin, Irina N. Babkina

**Affiliations:** 1 Laboratory of Molecular Microbiology, Institute of Chemical Biology and Fundamental Medicine, Novosibirsk 630090, Russia; 2 JSC VECTOR-BEST, Novosibirsk 630559, Russia; E-Mail: irinanik_babkina@mail.ru

**Keywords:** variola virus, orthopoxvirus, evolution, origin

## Abstract

The question of the origin of smallpox, one of the major menaces to humankind, is a constant concern for the scientific community. Smallpox is caused by the agent referred to as the variola virus (VARV), which belongs to the genus *Orthopoxvirus*. In the last century, smallpox was declared eradicated from the human community; however, the mechanisms responsible for the emergence of new dangerous pathogens have yet to be unraveled. Evolutionary analyses of the molecular biological genomic data of various orthopoxviruses, involving a wide range of epidemiological and historical information about smallpox, have made it possible to date the emergence of VARV. Comparisons of the VARV genome to the genomes of the most closely related orthopoxviruses and the examination of the distribution their natural hosts’ ranges suggest that VARV emerged 3000 to 4000 years ago in the east of the African continent. The VARV evolution rate has been estimated to be approximately 2 × 10^−6^ substitutions/site/year for the central conserved genomic region and 4 × 10^−6^ substitutions/site/year for the synonymous substitutions in the genome. Presumably, the introduction of camels to Africa and the concurrent changes to the climate were the particular factors that triggered the divergent evolution of a cowpox-like ancestral virus and thereby led to the emergence of VARV.

## 1. Introduction

Smallpox was a severe human disease caused by the variola virus (VARV), which was both highly lethal and highly contagious [1,2,3,4,5,6]. VARV is a member of the genus *Orthopoxvirus* [4,5,6]. A characteristic feature of this virus is its strict specificity for humans. This viral pathogen circulated in the human population for many centuries and caused repeated large-scale epidemics with great numbers of both recovered and dead victims [3,7]. In the 18th century, smallpox was the cause of death of more than four hundred thousand people in Europe each year [3]. Fortunately, this particular virus was completely eliminated from the human community by the end of the last century due to vaccination [3,4,8].

The closest relatives of VARV, which are also pathogenic to humans, are the Old World orthopoxviruses, including the vaccinia (VACV), cowpox (CPXV), and monkeypox (MPXV) [5,6]. The major natural reservoir of the last two viruses is rodents. For a long time, the introduction of laboratory strains made it impossible to detect wild VACV in nature [3,4,9,10,11]. It is currently believed that VACV originated from the horsepox virus (HPXV) [12]. However, all these viruses that are closely related to VARV have broad ranges of sensitive hosts; correspondingly, it is of paramount importance to clarify the factors that enhanced the evolutionary specialization of the VARV ancestor towards its single host, humans.

**Figure 1:**
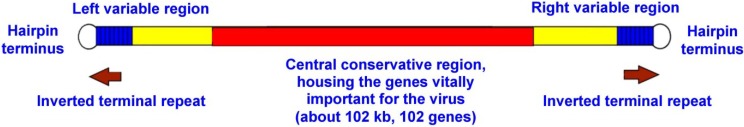
Scheme of orthopoxvirus genome (not to scale).

The major mechanisms in the evolution of poxviruses are the accumulation of random mutations and their subsequent fixation in the genome, gene amplification/reduction, and recombination [13,14,15]. The hereditary information of VARV is stored in a double-strand DNA molecule with a length of approximately 190 kbp (Figure 1). Similar to the other poxviruses, the VARV genome comprises an extended central conserved region (approximately 102 kbp), which primarily houses the vital genes responsible for protein modification, DNA repair, transcription, mRNA biosynthesis, replication, the structural components of the virions, and the variable terminal regions, which carry the genes for immunomodulatory proteins and the host range [5,16,17,18,19]. The accumulation of primarily random mutations is characteristic of the central conserved region in the VARV genome, whereas recombination events have been detected in the terminal genome regions [15]. Correspondingly, the phylogeny based on the VARV central conserved genome region most reliably reflects the evolutionary tree of this virus.

## 2. Smallpox in Ancient Times: Historical Data

The question of what caused smallpox was always intriguing and became the object for various legends and myths [3,7]. Despite the distinct characteristic features of smallpox that distinguish it from the other diseases, descriptions of this disease are absent from the written sources of ancient civilizations, such as the Bible and Talmud. Currently, most researchers regard smallpox descriptions in the *Charaka Samhita* and *Sushruta Samhita*, which are ancient Indian treatises, as the first reliable descriptions. These medical texts had been compiled by the 1st–4th centuries of the Common Era. Several sources have dated these medical texts to the 6th or even 15th century BC; however, we can only state that smallpox existed in India before Christ [7,20].

The first reliable smallpox description in China dates back to the 4th century AD, although there are numerous lines of evidence that smallpox was imported to China in the 3rd century BC [7]. There is a hypothesis that smallpox appeared in China in 1122 BC; however, this hypothesis is not confirmed by the majority of the historical evidence [3,7].

The most frequently referred to evidence about the putative spread of smallpox in ancient Egypt is associated with the mummy of Ramses V, who died in 1157 BC [3,7]. His mummy exhibits signs of skin lesions that were possibly caused by smallpox. Nonetheless, there is no historical evidence that reliably describes a smallpox epidemic in ancient Egypt. Such skin lesions might have been caused by various exanthematic diseases, including those caused by other poxviruses.

The available historical records allow for the assumptions that smallpox was imported to Greece during the second year of the Peloponnesian War in 430 BC, and was described as the “Plague of Athens”, and into Rome in 170 AD, where it was referred to as the “Antonine Plague”. However, unambiguous descriptions of smallpox in Europe are dated to only the 6th century AD [3,7,21,22].

All these historical data confirm the hypothesis that smallpox is a comparatively recently emerged infection. Indeed, VARV is transmitted only between sensitive persons and causes a highly lethal disease, but it does not infect animals. As a result of a smallpox epidemic, the majority of the sensitive population either becomes immune or dies, and the epidemic fades. Later, the virus might reappear in the same territory, but only after a sufficient number of new-generation of smallpox-sensitive children are born. Such a viral lifecycle requires a considerable concentration of sensitive hosts. Measles is another disease that is pathogenic only to humans and requires a population of approximately 200–300 thousand people for a sufficient number of sensitive children to appear and for the measles virus to be maintained in the human population. It is believed that smallpox shares similar characteristics. The majority of researchers assume that animal domestication, the development of land farming, and the establishment of large human settlements some 6000–10,000 years ago created the conditions that allowed the emergence of smallpox [7,23].

The high lethality rate of smallpox is another factor that favors a recent origin of VARV. This feature is frequently typical of viruses that have recently adapted to new hosts. In many cases, further evolution of such viruses leads to decreases in the case fatality rate [24,25,26]. An example of such evolution is the history of the application of the myxoma virus (genus *Leporipoxvirus*) to control the European rabbit (*Oryctolagus cuniculus*) population in Australia. In this case, the lethality rate of myxomatosis rapidly decreased [27,28,29]. The origin of the West African MPXV subtype is an additional illustration of the evolutionary strategy of orthopoxvirus. The emergence of this virus is dated to approximately 1200 years ago [30]. The loss of the complement-binding protein differentiated this subtype from the Central African MPXV strains and resulted in a reduction in the case fatality rate [31].

## 3. Dating VARV Evolution

Reconstructions of the evolutionary histories of viruses are based on comparisons of their nucleotide or amino acid sequences using the numbers of distinctions as a measure of evolutionary divergence [14,32]. The discovery of historical relics containing virus DNA would considerably enhance the dating of the evolutionary history of VARV. Researchers have attempted to isolate VARV DNA from various specimens [33]. The only successful attempt was that by Biagini *et al.* [34,35] who found VARV DNA in samples from corpses exhumed from permafrost. Despite a considerable degree of degradation, the extracted specimens were appropriate for sequencing and analyses of the structure of the ancient virus genome; however, this study is not yet complete. Consequently, the evolutionary history of VARV can be dated based on either the dates from which the VARV-containing samples were isolated or the assumed dates when the different VARV subtypes diverged from their ancestors.

It is known that smallpox was exported to Central and South America from West Africa in the early 16th century and caused high case fatality epidemics among the local population. Subsequent smallpox outbreaks in America had low lethality, and the virus that caused these epidemics acquired the name variola *minor alastrim* [3,7]. The evolution of the West African subtype of VARV, during its spread to the American continent, led to reduction in the case fatality rate of smallpox from 8%–12% to less than 1% [15].

Babkina *et al.* [36] analyzed restriction fragment length polymorphism (RFLP) data and discovered that the West African VARV strains belonging to the variola *major* subtype and the variola *minor alastrim* isolates composed a detached VARV biological subtype that exhibited essential distinctions in their genomic organization compared to the other VARV strains. Importantly, the West African and *alastrim* VARV strains clustered into two different phylogenetic branches, which led to the assumption of their independent evolution over a certain period of time. The phylogenetic relationships between VARV strains were later confirmed by sequencing of their genomes [15]. Based on data from RFLP analysis [36], Babkin assumed that variola *minor alastrim* originated from the West African VARV strains, and the first attempt to estimate the rate of poxvirus evolution was based on this assumption [37]. This analysis involved the central conserved region of the orthopoxvirus genomes and was conducted using a strict molecular clock and the maximum likelihood method. The results demonstrated that VARV diverged from the camelpox virus (CMLV) approximately 6000 years ago. The rate of substitution accumulation in the genomes of orthopoxviruses was estimated to be approximately 1 × 10^−6^ substitutions/site/year.

Li *et al.* [38] attempted to date the time of VARV emergence using other constraints. These authors only analyzed the SNPs that met the requirement of seven nucleotides surrounding an SNP and conserved nucleotides on both its sides. The complete orthopoxvirus genomes were studied in the evolutionary analysis described in this paper, which included the terminal variable regions of the genomes that were subjected to recombination [15] and the encoding of the virulence genes, many of which are under adaptive selection [16,17,39]. Two different assumptions about the time of VARV emergence were proposed by Li *et al.* The first analysis was based on the assumption that VARV was imported to the south of Africa in 1713 and then colonized the overall continent. However, there are numerous documented records of earlier smallpox spread on this continent; regarding North Africa, VARV was present there at least as early as the 7th century AD [3,7]. The second assumption was based on a description of smallpox in an ancient Chinese manuscript dated to the 4th century AD. Consequently, the authors inferred that VARV either emerged 16,000 years ago according to in the first case, or 68,000 years ago in the second case using a strict molecular clock. The authors suggested that smallpox appeared on the American continent long before Columbus discovered America. This contradicts the historical records that the American population was reduced by almost nine million people over 10 years of colonization due primarily to smallpox [3,7]. Note that VARV belongs to the Old World orthopoxviruses and is genetically distant from the New World orthopoxviruses [40]. Additionally, it is difficult to imagine that the population of VARV-sensitive hosts reached a sufficient density 68,000 years ago.

**Figure 2:**
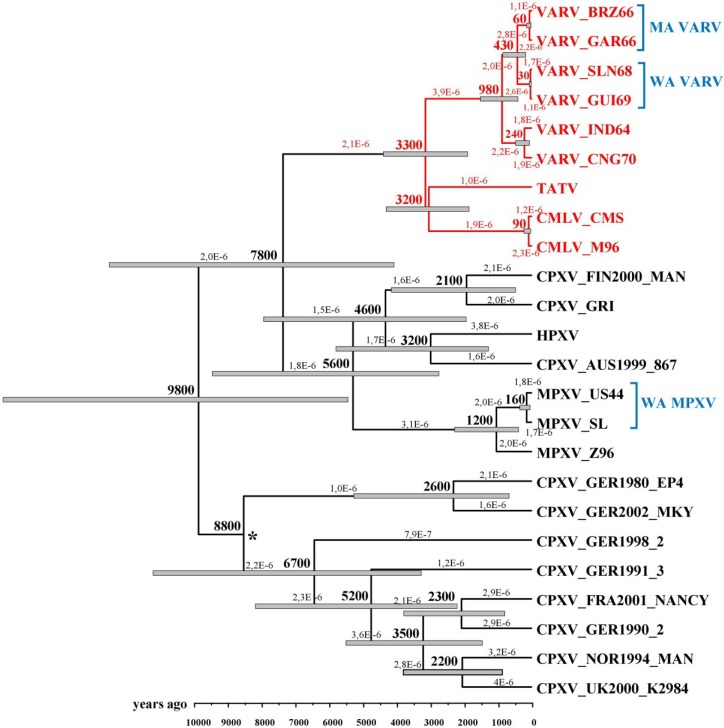
Chronogram of the maximum credibility tree for the orthopoxviruses generated with BEAST based on the central conserved regions of their genomes. The gray bars at the nodes represent the 95% highest probability density intervals. The rates of mutation accumulation are shown near the branches (substitutions/site/year). The numbers on nodes indicate the time to the most recent common ancestor of the clades (years ago). The posterior probabilities of all clades are >90% with the exception of the node marked with an asterisk. VARV—variola virus, TATV—taterapox virus, CMLV—camelpox virus, CPXV—cowpox virus, HPXV—horsepox virus, MPXV—monkeypox virus, MA VARV—variola *minor alastrim* strains, WA VARV—West African variola virus strains, WA MPXV—West African monkeypox virus strains. Legend and figure reproduced from [30].

In 2008 and 2012, Babkin *et al.* [30,41] analyzed the evolutionary history of VARV based on the conserved central regions of the orthopoxvirus genomes that comprise 102 genes with the help of a relaxed molecular clock. In the former work, the following constraints were used: the time period of the divergence of the VARV *alastrim* strains from the West African strains did not exceed 400 years, and the time of VARV emergence was less than 10,000 years ago. The results implied the possibility that the West African VARV subtype was imported to America not with the first slaves but later. The time scale for orthopoxvirus divergent evolution was assessed using a Bayesian dating method and Multidivtime software. The authors computed that the VARV, CMLV, and taterapox viruses (TATV) diverged from the same ancestor approximately 3400 years ago and estimated the evolution rate to be 2.3 × 10^−6^ substitutions/site/year. In the latter work, an expanded set of various orthopoxvirus strains was considered. Babkin *et al.* utilized Beast software and relaxed (uncorrelated lognormal) the molecular clock to assess the chronological scale. VARV emergence was estimated to have occurred approximately 3,300 years ago, and the rate of mutation accumulation was estimated to be 2.1 × 10^−6^ substitutions/site/year (Figure 2).

In 2010, Hughes *et al.* [42] analyzed the sequences of 132 orthopoxvirus genes and estimated the number of synonymous substitutions per synonymous site. Assuming the divergence of *alastrim* from the VARV West African strains occurred 300 to 500 years ago, these authors obtained substitution rates of 6 × 10^−6^ and 4 × 10^−6^ substitutions/site/year, respectively. Note that the separation of VARV from TATV was assessed at approximately 3000–4000 years ago, which matches previous data [30,41,43].

Firth *et al.* [44] applied a different method to estimate the evolution rates. These authors established the substitution accumulation rates in the genomes of different VARV strains based on the times of their isolations. They obtained slightly higher evolution rates than those reported in previous articles [30,41,42], which can be explained by the terminal highly variable regions of the genomes, which were used in the analysis [16,17,39].

Kerr *et al.* examined similar data about the evolution rates of another poxvirus, the myxoma virus [28]. The authors studied the attenuation of the myxoma virus following its introduction with the goal of achieving biological control of the European rabbit populations in Australia and Europe. The computed rates for myxoma virus evolution were two- to threefold higher than those of the orthopoxviruses, which is explainable by the rapid adaptation of this virus to its new host, the European rabbit.

One can conclude that most researchers have obtained similar orthopoxvirus evolutionary rates and dates of VARV origin using different approaches.

## 4. VARV Ancestor

Phylogenetic reconstructions suggest that CMLV, TATV, and VARV emerged from a common progenitor almost simultaneously [30,40,41,42,43,45,46]. Additionally, these viruses are strictly specific for their hosts, and both VARV and CMLV cause diseases with high case fatality rates [15,23]. A high density of susceptible hosts was necessary for the origin of these viruses [7,23,24]. It is known that the naked sole gerbil (*Gerbilliscus kempi*), which lives in the savannas and dry forests of Africa, is the only host for TATV, whereas the common gerbil (*Meriones unguncuilatus*) is not sensitive to TATV [47]. The fact that these three viruses are closely related suggests the existence of a common ancestral virus with a broad host range. Presumably, this virus affected rodents and was able to infect various representatives of the order *Rodentia* because the natural hosts of the majority of the current Old World orthopoxviruses (e.g., CPXV and MPXV) are hosts are rodents, but VARV and CMLV are exceptions [6,48]. The putative natural source of VACV is the horsepox virus, which is known to be pathogenic for rodents [48].

Presumably, a CPXV-like virus was the ancestor of VARV, TATV, and CMLV. It is known that CPXV has the broadest range of susceptible hosts and the longest genome of all known orthopoxviruses, and CPXV contains all of the orthopoxviral genes [17,19]. It is currently believed that all orthopoxviruses evolved from a CPXV-like progenitor via the shortening of the genome and mutations of some genes. These processes resulted in the emergence of narrower, specialized pathogens [18,39,46]. The CPXV strains are so genetically diverse that it has been suggested that they should be assigned to separate orthopoxvirus species [49,50].

## 5. Where Did VARV Emerge?

Researchers have tended to associate the site of VARV emergence with the first historical evidence describing smallpox and with the appearance of the first civilizations that produced large human settlements that allowed a new virus to emerge. All authors have indicated the Middle East and India as the putative geographic regions of VARV emergence [7,23,30,41,51].

Shchelkunov [51] assumed that VARV emerged in India. This assumption was based on the genetic variability of the different Indian VARV strains. Previously, it was noted that the Indian strains did not form a common cluster on the phylogenetic tree [52]. However, this fact could be explained by the large-scale smallpox epidemics in India in recent times and the subsequent spread of this virus to other Asian regions. Note that the genetic distances between the Indian strains are small on the phylogenetic tree. It should be emphasized that the most pronounced genetic difference was observed between the West African VARV subtype and the other VARV strains, which suggests an African origin of VARV [52]. Additionally, this hypothesis does not explain the origin of TATV because the naked sole gerbil, the host of TATV, does not inhabit India [53,54].

**Figure 3:**
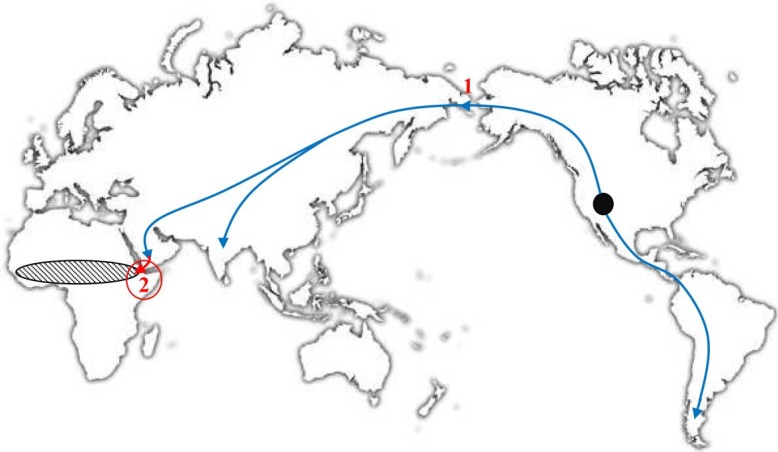
World map. The Black circle denotes the putative region of the origin of the *camelid* (*Camelidae*) ancestors approximately 45 MYA [60]. The direction of their migration is shown with arrows: 1, migration of the camel ancestors from North America to Asia 2–3 MYA [61]; and 2, introduction of domesticated camels into East Africa approximately 4 TYA [55,56]; hatched oval: the distribution area of naked sole gerbils [53,54]. Legend and figure reproduced from [30].

In 2012, Babkin and Babkina [30] proposed a hypothesis about the geographic region of VARV emergence that is based on the assumption that CMLV, TATV, and VARV originated at the same time and in the same geographic area. Indeed, as mentioned above, these three viruses originated from a common ancestral virus. Most probably, this virus was a CPXV-like virus that was able to infect rodents and other mammals. These authors studied the geographic ranges of the hosts of CMLV, TATV, and VARV. It is known that the naked sole gerbil is the only host of TATV [47], this rodent species is distributed from West Africa to Ethiopia, and its distribution range is confined by tropical forests in the south and the Sahara Desert in the north (Figure 3) [53,54]. Domesticated camels were imported for the first time to Africa, specifically, the Horn of Africa, 3500–4500 years ago, further advanced to Egypt in the 6th–7th century BC, and subsequently spread to other regions of the African continent [55,56]. There is evidence that large settlements existed approximately 4000 years ago in the Horn of Africa [57]. This geographic region might be the zone in which the distributions of the CMLV, TATV, and VARV hosts overlapped (Figure 3). Consequently, the putative area in which these three orthopoxvirus species emerged from a common progenitor might be the Horn of Africa. This hypothesis supports the dating of VARV emergence because the camel and naked sole gerbil did not meet in the same area before 4000 years ago. Babkin and Babkina [30] assumed that the evolution of a CPXV-like ancestral virus and its further separation into three highly specialized species were triggered by the introduction of the camel, a new potential host with unique antibodies [58,59], and the need for the virus to adjust to changing conditions.

The most recognized drivers of pathogen emergence are climate change, destruction of the environment of potential hosts, penetration of the pathogen into a new area, pathogen spread to other populations of hosts, and interactions with the host immune system [62]. A study of the global ecological factors that might have stimulated the emergences of CMLV, TATV, and VARV from the ancestral virus approximately 3500 years ago suggests that it is reasonable to consider the Santorini Eruption, which occurred between 1650 and 1540 BC [63,64]. This eruption was one of the largest volcanic events on Earth in recorded history and caused considerable climate changes [64] that presumably caused the migrations of various mammals and might have forced the evolution of the VARV ancestor. However, the coincidence of the timing of these climate changes and the emergence of VARV does not prove the relationship between these events.

## 6. The Molecular Evolution of VARV

Some authors have focused on the question of which particular changes in the genetic structure of the ancestral virus enable the emergence of VARV and the adaptation to humans. Rothenburg *et al.* [65,66] have thoroughly analyzed the host range genes in different poxvirus genomes, including that of VARV. The poxvirus genomes were comprehensively compared by several scientific teams [15,18,23,65]. The distinctions of the VARV genome from the genomes of the other orthopoxviruses were clarified. Smithson *et al.* [45] succeeded in detecting the mutation hotspot via comparisons of the genomes of VARV and other orthopoxviruses. This site corresponds to the O1L gene, which allows for the efficient replication of vaccinia virus in human cells. The O1L gene is not functional in the closely related CMLV and TATV viruses, which is suggestive of its important role in the adaptation of VARV to humans. Other researchers have studied the groups of VARV genes that are responsible for overcoming the host defense systems and compared these genes with the corresponding genes of other orthopoxviruses [67,68,69,70,71,72]. All of these works have, step by step, brought us closer to understanding the genetic mechanisms underlying the pathogenesis of smallpox and the adaptation of VARV to humans.

## 7. Conclusions

The phylogenetic and evolutionary studies of the genetic structures of orthopoxviruses that involved the historical records and epidemiological data suggest that VARV is a relatively young virus that emerged approximately 3000 to 4000 years ago in the east of Africa [30,41,42,44]. The introduction of camels to Africa 3500 to 4500 years ago with the considerable climate changes that occurred at this time might have triggered the evolution of a CPXV-like ancestral virus. In its initial stage, this progenitor virus was able to infect a wide range of hosts and presumably spread mainly through rodent populations before encountered the camel, which was a new species in Africa. Further divergent evolution due to subsequent adaptation of the ancestral virus to the new hosts, *i.e.*, the camel, naked sole gerbil, and human, led to emergence of three new highly specialized viruses: CMLV, TATV, and VARV. The evolutionary changes in the CPXV-like ancestral virus upon its encounter with the camel during a relatively short time span demonstrated that it is important to survey camels as a potential source of new zoonotic infections. 

The driving force of further research into VARV evolution might be the study of the VARV genomes of ancient specimens that are obtained, for example, from mummies buried in the permafrost. Such analyses would allow the evolutionary potential of the poxviruses to be estimated in greater detail. Currently, the emergence of a new poxvirus that is dangerous to humans remains a possibility.
